# Hyperleukocytosis during clozapine treatment: A rare presentation of B-cell Acute lymphoblastic leukemia

**DOI:** 10.1016/j.lrr.2021.100253

**Published:** 2021-05-31

**Authors:** Neslyne B Augustin, Michael Maroules

**Affiliations:** a Department of Internal medicine, St Joseph's University Medical Center, Paterson, NJ, United States of America; b Division of Hematology and Oncology, St Joseph's University Medical Center, Paterson, NJ, United States of America

**Keywords:** B-cell Acute lymphoblastic leukemia, Clozapine toxicity, Hyperleukocytosis, Schizophrenia

## Abstract

Clozapine has been widely used as an antipsychotic drug for the treatment of refractory schizophrenia. Unfortunately, a wide range of blood dyscrasias have been reported as adverse effects of this drug. Agranulocytosis has gotten the most clinical vigilance; however, there are substantial reports of other blood dyscrasias associated with Clozapine some more serious than others. Of relevance, there have been previous claims of Clozapine-associated leukocytosis and acute myeloid leukemia. We report the case of a 31-year-old patient who developed Acute lymphoblastic leukemia shortly after starting treatment with Clozapine for refractory schizophrenia. We suggest Clozapine may play a causal role in the development of leukemias in patients taking this medication and we encourage vigilance for such correlation.

## 1 Background

Acute lymphoblastic leukemia (ALL) is known to have a bimodal distribution with occurrence in children and in adults in the fifth decade of life. Incidence is twice as high in the children population than in the latter [5]. Studies of the adult population affected by ALL show a higher incidence in males and in Hispanics [9]. ALL is subcategorized into B-cell ALL or T-cell ALL depending on progenitor cells characteristics. B cell ALL (B-ALL) is a subtype of ALL that involves a dysregulation of B cell lymphoid progenitor cells. B-ALL is more common than the T cell subtype [5].

Clozapine is an atypical antipsychotic drug that has been shown to be superior to other drugs in the same class in patients with medication-resistant schizophrenia [10]. Agranulocytosis is a well-established side effect of this drug and is routinely monitored in patients taking this medication. Other blood dyscrasias get limited attention during Clozapine treatment [1].

There has been increasing evidence of cytotoxicity of Clozapine. More specifically, the metabolite N-desmethyl clozapine has been identified as toxic to myeloid precursor cells [4]. This raises the possibility of this metabolite being toxic to a more primitive stem cell precursor of both myeloid and lymphoid stem cells. Both Clozapine and N-desmethyl Clozapine were shown to be toxic to CD34+ progenitor cells. A study using the Danish register found an eight-fold increased risk of developing acute myeloid leukemia (AML) in patients treated with Clozapine versus patients not receiving clozapine [8]. In fact, cases of malignancies in patients receiving Clozapine for treatment-resistant schizophrenia have been substantial enough to generate ample literature discussion about the continuation of this medication during chemotherapy in that population [3]. Nonetheless, few of those investigations considered Clozapine as the potential carcinogenic agent. Association of clozapine with lymphoma has also been reported [7]. Toxicity has been reported in other organs; we cite clozapine-associated myocarditis [6] and parenchymal lung disease [2]. The latter is potentially conflicting with the finding of lower incidence of lung cancer among patients taking clozapine [11]. Studies on both sides do agree that Clozapine has considerate cellular effects commendable for further study.

We report the case of a patient with medication-resistant Schizophrenia who developed B-ALL shortly after starting Clozapine treatment.

## 2 Case presentation

The case is about a 31-year-old Hispanic male with Schizophrenia diagnosed at age 29. The patient had been treated with multiple medication regimens without much success to control his psychotic outbreaks. The patient was most recently started on clozapine by his psychiatrist and was subsequently scheduled for regular blood monitoring. The patient had been taking Clozapine for a total of 8 months. Routine complete blood count (CBC) was repeated every two weeks. White blood cells (WBC) trended from 6.9 x10³/μL to 21.2 x10³/μL in two weeks. On the next routine CBC, WBC spiked to 104x10³/μL. (See Table 1*)* This prompted the psychiatrist to send the patient to Emergency department for further evaluation.

**Table 1:** Ten-week trend CBC with differentials.

	DOA*	2-wks	4-wks	6-wks	8-wks	10-wks
**WBC (x10³/μL)**	104.0	21.2	6.9	5.2	4.3	5.8
Neutrophils (%)	4	2	20	29	41	43
Bands (%)	2	3	3	0	1	3
Lymphocytes (%)	10	65	48	70	50	51
Monocytes (%)	1	1	3	1	8	2
Eosinophils (%)	2	0	0			
Basophils (%)	0	0	0			1
Metalymphocytes (%)	0	1	1			
Blast (%)	3	15	7			
Atypical lymphocytes (%)	78	13	19			
**Hemoglobin (g/dL)**	12.1	14.4	15.1	14.9	14.5	14.6
**Platelets (x 10³/μL)**	104	132	190	263	273	289

-wks: weeks before DOA.

⁎ DOA: day of admission.

Upon admission, review of systems was positive only for vague generalized weakness and fatigue for the past “few” months. Patient denied any fever, chills, night sweats, weight loss, sore throat, cough, chest pain, SOB, abdominal pain, nausea, vomiting, or changes in bowel movements. He also denied any rash or bleeding from his gums. There is no family history of malignancy. Physical exam was significant only for obesity and tender cervical lymphadenopathy noted on the right.

Laboratory was significant for hyperleukocytosis with WBC 104x10³/μL (normal range: 4.5–11x10³/μL). Differentials were 78% atypical lymphocytes, 10% lymphocytes, 3% blasts (Figure 1), 4% neutrophils, 2% bands, 1% monocytes, 2% eosinophils (*See Table 1 for 10-week trend).* Laboratory was also significant for mildly elevated LDH 289 unit/L (normal range: 140-271 unit/L) and new-onset thrombocytopenia and normocytic anemia. HIV was negative. Peripheral blood Flow cytometry analysis showed 85% of the cells consisting of TdT+, CD34+ B cell lymphoblasts; consistent with the diagnosis of B cell ALL (*See* Table 2*).*

**Table 2:** Peripheral blood flow cytometry analysis.

**Viability & Abnormal Cells**	
Viability	89%
Anormal Cells	Yes
% Abnormal Cells	85%
Cell Size	Large
**Cell Distribution**	
Lymphocyte gate	7.67%
Blast Gate	90.47%
Monocyte Gate	0.48%
Granulocyte/Myeloid gate	1.24%
Erythroid/Plasma Cell Gate	0.00%

B-lymhoblasts (TdT+, CD79a+, CD34+, CD19+, CD20dim+, CD22+) with co-expression of CD10 and HLA-DR.

The mature B-cells (1.1% of total) appear polytypic.

The T-cells (4.4% of total) show no pan T-cell antigenic deletion.

CD4: CD8 T-cell ratio is 1:1.

## 3 Treatment

Soon after medication review during admission, Clozapine was discontinued. Patient was placed on a pediatric-inspired protocol given his age and poor prognostic feature including WBC>30x10³/μL . He received induction with Hyper-CVAD regimen. He was positive for minimal residual disease (MRD). Patient later relapsed and was then started on Inotuzumab after which he achieved remission.

## 4 Discussion

This case offers the peculiar development of hyperleukocytosis during Clozapine treatment which is paradoxical to the more commonly anticipated agranulocytosis. This possibly explains the delay in clinical investigation even after the CBC revealed WBC 21 x10³/μL two weeks prior to admission; such high values were already within investigative range. Also significant with these values were 15% blasts and 13% atypical lymphocytes.

We should point this patient's age of 31 falls outside the more common population of the bimodal distribution of ALL. On the other hand, his Hispanic origin places him in the high-risk lifetime incidence population.

Our case support previous claim that Clozapine may increase the risk of hematologic malignancies. We also believe correlation of clozapine with such malignancies may have been missed. We encourage report of similar cases for improve awareness during usage of this drug.

## References

[1] Alvir, Jose M.J., Lieberman Allan ZS, Schwimmer Jeffrey L, Schaaf John A (1993). Clozapine-induced Agrnulocytosis - Incidence and risk factors in the United States. N. Engl. J. Med..

[2] Bugge E., Nissen T., Wynn R. (2016). Probable clozapine-induced parenchymal lung disease and perimyocarditis: a case report. BMC Psychiatry.

[3] Cunningham N.T., Dennis N., Dattilo W., Hunt M., Bradford D.W. (2014). Continuation of clozapine during chemotherapy: a case report and review of literature. Psychosomatics.

[4] Deliliers G.L., Servida F., Lamorte G., Quirici N., Soligo D. (1998). In vitro effect of clozapine on hemopoietic progenitor cells. Haematologica.

[5] Jabbour E., O’Brien S., Konopleva M., Kantarjian H. (2015). New insights into the pathophysiology and therapy of adult acute lymphoblastic leukemia. Cancer.

[6] Layland J.J., Liew D., Prior D.L. (2009). Clozapine-induced cardiotoxicity: a clinical update. Med. J. Aust..

[7] Leung J.G., Barreto J.N., Thompson C.A. (2018). Lymphoma following clozapine exposure: more information needed. Schizophr. Res..

[8] Nielsen J., Boysen A. (2010). Clozapine treatment associated with increased risk of acute myeloid leukemia (AML). Schizophr. Res. 2010.

[9] Pullarkat S.T., Danley K., Bernstein L., Brynes R.K., Cozen W. (2009). High lifetime incidence of adult acute lymphoblastic leukemia among Hispanics in California. Cancer Epidemiol. Biomark. Prev..

[10] Raguraman J., Vijay Sagar K.J., Chandrasekaran R (2005). Effectiveness of clozapine in treatment-resistant schizophrenia. Indian J. Psychiatry.

[11] Yin Y.C., Lin C.C., Chen T.T. (2015). Clozapine induces autophagic cell death in non-small cell lung cancer cells. Cell Physiol. Biochem..

